# Latent Class Analysis of Shift Work Patterns Among Nurses and Its Impact on Anxiety–Depression Comorbidity: A Multicenter Cross‐Sectional Study

**DOI:** 10.1155/da/8160605

**Published:** 2026-07-30

**Authors:** Chen Zhao, Xuezhi Shi, Meng Fan, Min Huang, Yajuan Yang, Yaqian Niu, Miao Zhang, Wendi Xu, Yapeng Deng, Mei Jin, Xinyu Hu, Ying Tang, Minmin Jiang, Fangbiao Tao, Heping Wang, Xiaoyan Wu

**Affiliations:** ^1^ School of Nursing, Anhui Medical University, Hefei, Anhui, China, ahmu.edu.cn; ^2^ Department of Nursing, Lu’an People’s Hospital, Lu’an, Anhui, China, layy.cn; ^3^ Department of Otolaryngology-Head and Neck Surgery, Guangdong Provincial People’s Hospital, Guangzhou, Guangdong, China, gdghospital.org.cn; ^4^ Department of Occupational Health and Environmental Health, School of Public Health, Anhui Medical University, Hefei, Anhui, China, ahmu.edu.cn; ^5^ Department of Rehabilitation Medicine, Lu’an People’s Hospital, Lu’an, Anhui, China, layy.cn; ^6^ Department of Cardiology, The Second Affiliated Hospital of Wannan Medical University, Wuhu, Anhui, China; ^7^ School of Nursing, Anhui Medical University and Taikang Health and Wellness Industry Research Institute, Hefei, Anhui, China; ^8^ Department of Maternal, Child and Adolescent Health, School of Public Health, Anhui Medical University, Hefei, Anhui, China, ahmu.edu.cn; ^9^ Key Laboratory of Population Health Across Life Cycle (Anhui Medical University), Ministry of Education/Anhui Provincial Key Laboratory of Environment and Population Health Across the Life Course/NHC Key Laboratory of Study on Abnormal Gametes and Reproductive Tract, Anhui Medical University, Hefei, Anhui, China, ahmu.edu.cn; ^10^ Department of Nursing, The Second Affiliated Hospital of Wannan Medical University, Wuhu, Anhui, China

## Abstract

**Objective:**

Relatively few population‐based studies have explored the relationship between shift work patterns and anxiety–depression comorbidity among nurses. This study aimed to identify different patterns of nurse shift work and assess their association with anxiety–depression comorbidity.

**Design:**

This study was based on the Nurses’ Environmental and Occupational Exposure and Health Nexus (NEON) cohort. A self‐designed questionnaire was used to collect general demographic information and shift work details. Anxiety and depressive symptoms were evaluated using the generalized anxiety disorder‐7 (GAD‐7) and the patient health questionnaire‐9 (PHQ‐9), respectively. Latent class analysis (LCA) was performed to identify homogeneous groups of shift work. A three‐step approach of LCA, involving logistic regression models, was applied to examine associations between various shift patterns and anxiety–depression comorbidity. Sleep quality served as a stratification factor for the subgroups.

**Results:**

Three latent classes were identified among 2791 shift nurses: Class 1: high‐frequency, high‐seniority group (*N* = 441, 15.80%); Class 2: low‐frequency, low‐seniority group (*N* = 835, 29.92%); Class 3: low‐frequency, high‐seniority group (*N* = 1515, 54.28%). Binary logistic regression analysis showed that, compared with Class 2, Class 3 had increased risks of anxiety, depression, and comorbid anxiety and depression by 54.8% (OR = 1.548, 95% CI 1.304–1.838), 30.6% (OR = 1.306, 95% CI 1.096–1.555), and 47.6% (OR = 1.476, 95% CI 1.245–1.749), respectively. Moreover, Class 1 had the highest risk of these conditions in both unadjusted and adjusted models. Sleep quality had no significant modifying effect.

**Conclusions:**

This study identified distinct patterns in nurses’ shift work. Different patterns were significantly associated with anxiety–depression comorbidity. Targeted interventions addressing specific shift patterns may improve nurses’ mental health and contribute to achieving broader health goals.

## 1. Introduction

In the world, 3%–9% of adults have depression and 14% have anxiety. Among them, women have a higher risk [1, 2]. The nursing profession, due to its long‐term exposure to highly demanding and stressful working conditions, has particularly prominent mental health issues [3–5]. The prevalence of anxiety and depressive symptoms among nurses in China exceeds 58% and 62%, respectively [6]. The coexistence of both symptoms is defined as anxiety–depression comorbidity [7]. Notably, a study from Hong Kong, China, found that the prevalence of comorbid anxiety and depression among 850 nurses was 27.6% [7]. Comorbidity frequently results in more severe clinical symptoms and poorer prognosis. Therefore, identifying anxiety–depression comorbidity may be more practical than examining anxiety or depression alone.

Mental health issues among nurses have attracted widespread attention from scholars globally [4, 8]. Nurses’ mental wellbeing can be impacted by multiple factors, including age, occupational stress, pro‐inflammatory cytokines, insufficient sleep, and shift work [9–14]. Shift work is defined as a work schedule that is outside the conventional 8 h day [15]. Approximately 70.1% of healthcare professionals in China have shift work experience [11]. Evidence indicates that shift work leads to adverse outcomes among nurses, including circadian rhythm disruption, sleep disturbances, and occupational burnout [3–5]. Multiple studies have demonstrated a significant association between shift work and symptoms of anxiety and depression. A cohort study examining the relationship between shift work and anxiety–depression among Dutch workers found that the self‐reported levels of anxiety and depression symptoms among shift workers were higher than those of nonshift workers [16]. Moreover, a study using data from American shift workers showed that shift work increased the risk of depressive symptoms by 33% [17]. Although there have been studies exploring the relationship between shift work and the mental health of nurses, most of these studies on the nursing population have only focused on the impact of a single shift characteristic on a single psychological symptom [11, 18, 19]. Such isolated approaches risk underestimating or distorting the true impact on health. In real‐world clinical environments, shift work encompasses various dimensions, including shift work duration, shift frequency, and shift rotation order. Different shift dimensions may interact with each other [20], leading to increased mental fatigue and psychological vulnerability [5, 21]. A study conducted in Canada indicated that greater irregularity in shift scheduling was associated with a stronger link to depressive symptoms [22]. Therefore, considering the complexity of shift work allows for a more comprehensive understanding of its impact on health.

Latent class analysis (LCA) is a person‐centered analytical approach that captures population heterogeneity by identifying distinct subgroups based on shared characteristics [23]. To the best of our knowledge, no previous study has holistically examined the patterns of nurses’ shift work or explored how these patterns might be differentially associated with anxiety–depression comorbidity. Therefore, this study aimed to employ a more nuanced approach using LCA, focusing on latent classes of nurse shift work and examining the associations between different shift classes and comorbid anxiety and depression. This would provide a basis for developing intervention strategies tailored to each shift class and promote nurses’ mental health.

## 2. Methods

### 2.1. Setting and Study Design

This is a representative cross‐sectional study of Anhui Province based on the Nurses’ Environmental and Occupational Exposure and Health Nexus (NEON) cohort, enrolling nurses who completed baseline surveys between July 2022 and January 2024 as participants. The NEON cohort was officially established in 2022 and has conducted follow‐up visits every 2 years, aiming to longitudinally monitor the health status of the nursing profession. Participants were recruited using convenience sampling and multistage cluster sampling methods. First, six tertiary hospitals were randomly selected from Anhui Province (covering southern, northern, and central Anhui). Then, all female nurses who met the criteria in each selected hospital were enrolled through convenience sampling. The inclusion and exclusion criteria were as follows: (1) inclusion criteria: female nurses with a valid nursing practice certificate who provided informed consent and voluntarily participated; (2) exclusion criteria: retired nurses, intern nurses, those on leave or pregnant during the survey period, or those lost to follow‐up or unable to complete the questionnaire for any reason. The survey was administered using a web‐based platform (e.g., Wenjuanxing) to ensure anonymity and encourage honest responses. Before starting, a standardized instruction page was presented to all participants, clearly stating the research objectives, the voluntary nature of participation, the confidentiality of data, and definitions of key terms (e.g., shift work and night shift). To ensure data quality, the following quality control (QC) measures were implemented: (1) all questionnaire items were set as mandatory to prevent missing data; (2) one attention check question (e.g., ‘for this item, please select “A"’) was embedded in the questionnaire; respondents who failed this check were excluded from the final analysis; and (3) after data collection, two independent researchers screened the data for logical errors and multivariate outliers. The Ethics Committee of Anhui Medical University approved the study protocol, and all participants provided written informed consent. The Ethical Approval Number for this research is 83220409.

In this study, 4520 electronic questionnaires were collected, of which 500 were excluded as invalid. Of these, 83 (1.8%) contained errors in the QC questions, 261 (5.8%) could not be matched to personal information, and 156 (3.5%) were duplicate submissions. Consequently, 4020 valid questionnaires were ultimately completed for an effective response rate of 88.3%. Among them, 1229 nurses reported never working shifts, while the remaining 2791 individuals who reported currently engaging in shift work were ultimately included in the model analysis.

### 2.2. Exposure

A self‐designed questionnaire was used to investigate the current status of shift work among nurses. The survey first ascertained whether the nurses were currently working shifts; subsequently, those who responded affirmatively were further assessed shift rotation order, shift duration, night shift duration, small night frequency, and large night frequency. The shift work duration was divided into three equal groups according to tertiles: ≤6 years, 6.1–11 years, and >11 years. The frequencies of evening shifts and night shifts were categorized based on the median values as <6 times/month and ≥6 times/month, respectively. In this context, shift rotation order refers to the sequence in which workers rotate across different shifts (e.g., day, evening, and night). A clockwise rotation was defined as morning to evening to night, and a counterclockwise rotation as night to evening to morning; if it does not belong to either of the two, it is classified as other. Shift duration and night shift duration were both defined as the period from the start of shift work/night shifts to the date of questionnaire completion.

### 2.3. Outcome

Generalized anxiety disorder‐7 (GAD‐7) was used to assess anxiety symptoms in nurses. The GAD‐7 is a self‐reported scale consisting of seven items designed to screen and assess symptoms of GAD [24]. Responses to each item are classified into four levels, reflecting the severity of symptoms, with scores ranging from 0 (not at all) to 3 (nearly every day). The GAD‐7 total score ranges from 0 to 21, with higher scores indicating more severe anxiety symptoms among nurses. Based on previous studies, anxiety was defined as a GAD‐7 score of ≥5. The instrument demonstrates strong internal consistency (Cronbach’s *α*: 0.96).

The patient health questionnaire‐9 (PHQ‐9) was used to measure the prevalence of depressive symptoms, as it is designed to screen and assess depressive symptoms [25]. Responses to each item are classified into four levels, reflecting the severity of symptoms, with scores ranging from 0 (not at all) to 3 (nearly every day). The PHQ‐9 total score ranges from 0 to 27, with higher scores indicating more severe depressive symptoms among nurses. According to the recommended cutoff, participants with a score of ≥5 were considered at risk for depressive symptoms in our study. The instrument demonstrates strong internal consistency (Cronbach’s *α*: 0.92).

### 2.4. Confounders

Relevant confounding factors were identified through a literature review. General demographic information was collected using a self‐designed questionnaire, including department, age, educational level, marital status, number of children, medical history, professional title, annual personal income, sleep quality, and body mass index (BMI).

Sleep quality over the past month was assessed using the Pittsburgh sleep quality index (PSQI). This scale comprises 19 items and seven dimensions (sleep quality, sleep duration, sleep latency, sleep efficiency, sleep disorders, hypnotic drugs, and daytime function). Each component was scored on a scale of 0–3. The sum of the scores from the seven dimensions constitutes the global PSQI score, which ranges from 0 to 21, with higher scores indicating poorer sleep quality. Sleep quality was classified as good where the PSQI total score is ≤ 7 and as poor where the PSQI total score is >7. The instrument demonstrates strong internal consistency (Cronbach’s *α*: 0.73).

Height and weight of female nurses were collected through a questionnaire. The BMI was calculated as weight in kilograms divided by the square of height in meters (kg/m^2^).

### 2.5. Statistical Analysis

All data were analyzed using SPSS 23.0 and Mplus 8.11. We only included the cases of shift nurses (*N* = 2791). First, the five components of shift work were classified as present or absent (categorical indicator variables coded as 1 or 0) and analyzed using LCA.

We applied LCA to identify whether there were differences in nurses’ shift patterns. In our study, we fitted one to four latent class models to determine the optimal number of latent classes. Model fit indices used for the LCA included log‐likelihood (LL), Akaike information criterion (AIC), Bayesian information criterion (BIC), adjusted BIC (aBIC), Lo‐Mendell‐Rubin adjusted likelihood ratio (LMR), bootstrap likelihood ratio test (BLRT), and entropy [26]. Entropy is an indicator of classification accuracy, with values close to 1 indicating greater accuracy. This study identified a three‐class model as optimal based on the fit indices.

Second, after determining the appropriate number of latent classes, nurses were assigned to the most likely subgroups based on their highest posterior class probabilities during their shifts. The chi‐square test was used to examine the distribution of relevant risk factors. Subsequently, binary logistic regression was performed to analyze the association between latent classes of nurse shift work and comorbid depression and anxiety. The significance level was set at *p*  < 0.05.

Finally, although it is known that shift work disrupts sleep and sleep quality is likely to be located on the causal path between shift work and mental health, given the cross‐sectional nature of the data in this study and the lack of temporal sequence, we were unable to establish a causal relationship or distinguish between mediating and moderating effects. Therefore, we conducted subgroup analyses to explore whether sleep quality moderates the association between shift work patterns and mental health outcomes. The subgroups were stratified by sleep quality. The *p*‐value of the interaction was calculated through interaction tests. If the *p*‐value of the interaction exceeded 0.05, it indicated that no effect modification was found [27].

## 3. Results

### 3.1. LCA Results

**Figure 1:**
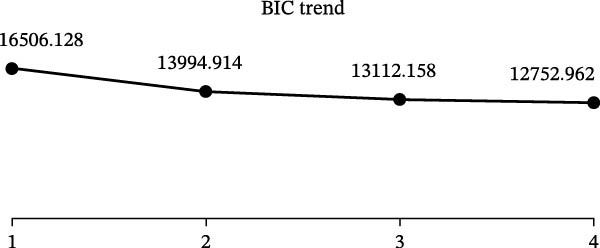
The steepness graphs of the BIC parameters for each model are displayed in the latent class analysis.

In this study, 2791 shift nurses were included for modeling. Five items regarding shift work from a self‐designed questionnaire were used as manifest indicators, and all variance inflation factor (VIF) values were less than 5. The model estimates 1–4 classes. Variable assignments are shown in Table 1, and model fit parameters are presented in Table 2. The entropy values for all models were greater than 0.8, and both the VLMR and BLRT were statistically significant (*p* < 0.05). As the number of categories increases, the values of AIC, BIC, and aBIC decrease accordingly. After three categories, the decline in BIC becomes relatively gentle (Figure 1). Therefore, the best models were selected for the three categories, indicating that there might be three subcategories of nurses’ shift work situations (Table 2).

**Table 1:** Variable assignment for latent classes model.

Manifest variables	Assignments
Small night frequency	<6 = 1, ≥6 = 2
Large night frequency	<6 = 1, ≥6 = 2
Shift duration	≤6 = 1, >6 = 2
Night shift duration	≤6 = 1, >6 = 2
Shift rotation order	Clockwise or counterclockwise = 1, other = 2

**Table 2:** Indexes for fitting the model of LCA in research competence (*N* = 2791).

Number of profiles	LL	AIC	BIC	ABIC	Entropy	BLRT	VLMR	Latent class probability (%)
						*p*	*p*	
1 class	8233.229	16476.458	16506.128	16490.242	—	—	—	—
2 class	6953.819	13929.638	13994.914	13959.963	0.992	<0.001	<0.001	67.50/32.50
**3 class**	**6488.639**	**13011.278**	**13112.158**	**13058.144**	**0.986**	<**0.001**	<**0.001**	**15.80/29.92/54.28**
4 class	6285.238	12616.476	12752.962	12679.883	0.976	<0.001	<0.001	9.961/7.381/28.38/54.28

*Note:* Bold values indicate the best models were selected for the three categories, indicating that there might be three subcategories of nurses’ shift work situations.

Abbreviations: ABIC, adjusted Bayesian information criterion; AIC, Akaike information criterion; BIC, Bayesian information criterion; BLRT, bootstrap likelihood ratio test; LL, log‐likelihood; LMR, Lo‐Mendell‐Rubin adjusted likelihood ratio.

### 3.2. Definition of Latent Class

**Figure 2:**
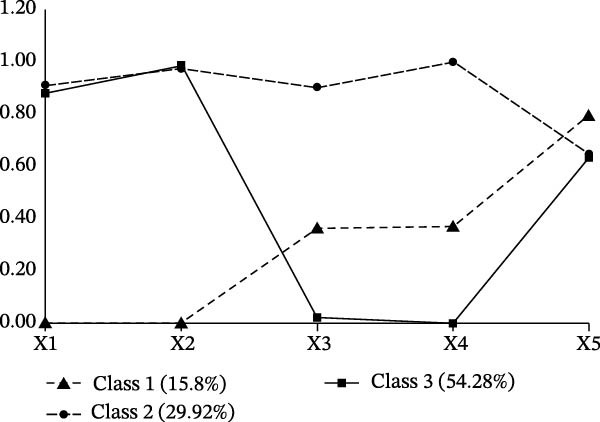
Response patterns for latent classes.

As shown in Table 2, the three potential classes are included: Class 1 (*N* = 441, 15.80 %), Class 2 (*N* = 835, 29.92 %), and Class 3 (*N* = 1515, 54.28%). The probability of latent class response yes is shown in Figure 2: (1) high‐frequency, high‐seniority group (Class 1): the frequencies of small and large night shifts were almost ≥ 6 times per month (99% and 100%, respectively); the probabilities of having >6 years of shift work and >6 years of night shift work were high (63.4% and 63.0%, respectively); and the shift rotation order was mostly clockwise or counterclockwise (79.7%). Therefore, this class was classified as the “high‐frequency, high‐seniority group.” (2) Low‐frequency, low‐seniority group (Class 2): the frequencies of small and large night shifts were almost <6 times per month (91.2% and 97.2%, respectively); the probabilities of having ≤6 years of shift work and ≤6 years of night shift work were extremely high (90.4% and 100%, respectively); and the shift rotation order was mostly clockwise or counterclockwise (64.8%). Therefore, this class was classified as the “low‐frequency, low‐seniority group.” (3) Low‐frequency, high‐seniority group (Class 3): the frequencies of small and large night shifts were almost <6 times per month (88.0% and 98.5%, respectively); however, the probabilities of having > 6 years of shift work and >6 years of night shift work were almost 100% (97.8% and 100%, respectively); and the shift rotation order was mostly clockwise or counterclockwise (63.4%). Therefore, this class was classified as the “low‐frequency, high‐seniority group.” All three categories are mainly based on regular shifts (clockwise/counterclockwise), with the differences lying in the combination of night shift intensity and duration of shift. Thus, the above naming can clearly reflect the shift characteristics of each subgroup.

### 3.3. Univariate Analysis of Latent Class of Depression

A total of 2791 participants were included in the analysis. The distribution of demographic characteristics across different shift groups is shown in Table 3. The results indicated that all variables differed significantly among the three classes (*p* < 0.05). Most participants had a normal BMI (70.6%) and no history of disease (65.9%). Class 3 had significantly higher numbers of participants with anxiety, depression, and anxiety–depression comorbidity than the first two groups.

**Table 3:** Characteristics of respondents across latent classes.

Characters	Detection results [*n* (%)]				*χ* ^2^	*p*‐Value
	Population (*N* = 2791)	Class 1 (*N* = 441)	Class 2 (*N* = 835)	Class 3 (*N* = 1515)		
Age						
<30	1286	235 (18.3)	775 (60.3)	276 (21.5)	1216.58	**<0.001**
≥31	1505	206 (13.7)	60 (4.0)	1239 (82.3)	—	—
BMI						
Normal	1970	304 (15.4)	574 (29.1)	1092 (55.4)	84.99	**<0.001**
Light	289	45 (15.6)	147 (50.9)	97 (33.6)	—	—
Overweight	431	75 (17.4)	90 (20.9)	266 (61.7)	—	—
Obesity	101	17 (16.8)	24 (23.8)	60 (59.4)	—	—
Education						
Junior college and below	962	153 (15.9)	359 (37.3)	450 (46.8)	42.11	**<0.001**
Bachelor’s degree or above	1829	288 (15.7)	476 (26.0)	1065 (58.2)	—	—
Annual income, CNY						
≤50,000	436	62 (14.2)	214 (49.1)	160 (36.7)	106.21	**<0.001**
60,000–100,000	1442	254 (17.6)	398 (27.6)	790 (54.8)	—	—
>100,000	913	125 (13.7)	223 (24.4)	565 (61.9)	—	—
Diseases history						
No	1840	297 (16.1)	591 (32.1)	952 (51.7)	15.58	**<0.001**
Yes	951	144 (15.1)	244 (25.7)	563 (59.2)	—	—
Sleep quality					—	—
Poor	1337	233 (17.4)	366 (27.4)	738 (55.2)	10.24	**0.006**
Good	1454	208 (14.3)	469 (32.3)	777 (53.4)	—	—
Anxiety						
No	1103	160 (14.5)	392 (35.5)	551 (50.0)	27.49	**<0.001**
Yes	1688	281 (16.6)	443 (26.2)	964 (57.1)	—	—
Depressed					—	—
No	974	136 (14.0)	331 (34.0)	507 (52.1)	12.83	**0.002**
Yes	1817	305 (16.8)	504 (27.7)	1008 (55.5)	—	—
Comorbidity of anxiety and depression						
No	1288	188 (14.6)	443 (34.4)	657 (51.0)	22.94	**<0.001**
Yes	1503	253 (16.8)	392 (26.1)	858 (57.1)	—	—

*Note*: Class 1, high‐frequency, high‐seniority group; Class 2, low‐frequency, low‐seniority group; Class 3, low‐frequency, high‐seniority group. Bold values indicate statistical significance.

Abbreviations: BMI, body mass index; CNY, China Yuan.

### 3.4. Association Between Shift Patterns and Anxiety–Depression Comorbidity

As shown in Table 4, shift latent classes were associated with anxiety, depression, and anxiety–depression comorbidity (*p*  < 0.05). In the unadjusted models, the low‐frequency, high‐seniority group (Class 3) and the high‐frequency, high‐seniority group (Class 1) were more likely to suffer from anxiety, depression, or anxiety–depression comorbidity than the low‐frequency, low‐seniority group (Class 2). Among these, the high‐frequency, high‐seniority group (Class 1) had the highest risk. In the models for anxiety and anxiety–depression comorbidity, adjusting for potential confounders partially attenuated these results, but the risk generally remained elevated with increasing shift frequency and duration of shift work (anxiety: Class 3 OR 1.443, 95% CI 1.129–1.844; Class 1 OR 1.504, 95% CI 1.159–1.951. Anxiety–depression comorbidity: Class 3 OR 1.365, 95% CI 1.072–1.738; Class 1 OR 1.457, 95% CI 1.128–1.881).

**Table 4:** Logistic regression analysis of latent classes of shift.

Variable	Anxiety		Depression		Anxiety–depression comorbidity	
	Model 0 OR (95% CI)	Model 1 OR (95% CI)	Model 0 OR (95% CI)	Model 1 OR (95% CI)	Model 0 OR (95% CI)	Model 1 OR (95% CI)
Low‐frequency, high‐seniority group (Class 3)	1.548 ^∗∗^ (1.304–1.838)	1.443 ^∗^ (1.129–1.844)	1.306 ^∗^ (1.096–1.555)	1.318 ^∗^ (1.026–1.695)	1.476 ^∗∗^ (1.245–1.749)	1.365 ^∗^ (1.072–1.738)
High‐frequency, high‐seniority group (Class 1)	1.554 ^∗∗^ (1.226–1.970)	1.504 ^∗^ (1.159–1.951)	1.473 ^∗^ (1.153–1.882)	1.474 ^∗^ (1.126–1.930)	1.521 ^∗∗^ (1.205–1.919)	1.457 ^∗^ (1.128–1.881)

*Note*: The low‐frequency, low‐seniority group (Class 2) served as the reference group for all comparisons. Model 0 did not adjust for any variables; Model 1 was adjusted for demographic variables: department, age, education level, marital status, number of children, history of disease, professional title, and personal annual income.

Abbreviations: CI, confidence interval; OR, odds ratio.

^∗^ *p* < 0.05.

^∗∗^ *p* < 0.01.

### 3.5. Subgroup Analysis

**Figure 3:**
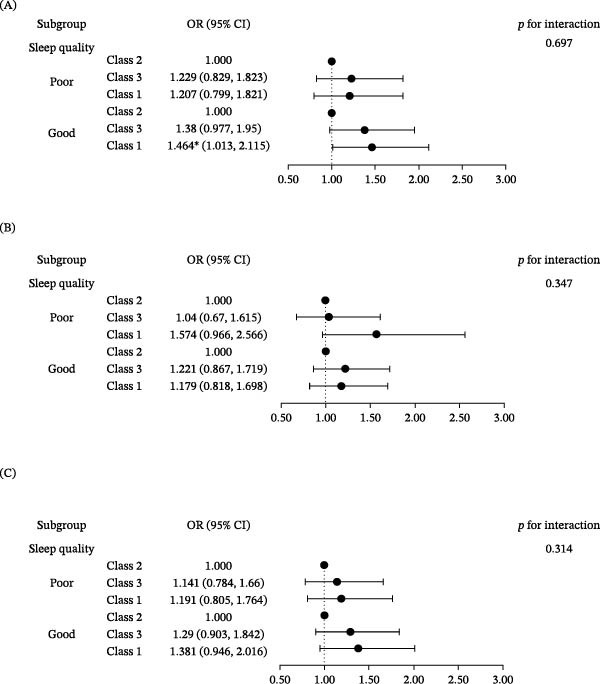
Forest plots of subgroup analyses. (A) Anxiety, (B) depression, and (C) comorbidity of anxiety and depression.  ^∗^ *p* < 0.05. Adjusted for demographic variables: department, age, education level, marital status, number of children, disease history, professional title, and personal annual income. Class 1, high frequency, high‐seniority group; Class 2, low‐frequency, low‐seniority group; Class 3, low‐frequency, high‐seniority group. CI, confidence interval; OR, odds ratio.

Numerous studies have confirmed that sleep quality plays a significant role in anxiety, depression, and comorbid anxiety–depression conditions [28–33]. In this study, participants were stratified by sleep quality in subgroup analyses (as shown in Figure 3). To test whether sleep quality modified the associations between shift work latent classes and the outcomes, we calculated *p*‐values for the interaction. The interaction *p*‐values for all three outcomes were >0.05, indicating no significant effect modification by sleep quality. However, when examining associations within each sleep quality stratum (as shown in the results for anxiety in Figure 3A), a significant association with anxiety was observed in Class 1 within the good sleep quality stratum (marked with  ^∗^), whereas no significant association was observed in the poor sleep quality stratum.

## 4. Discussion

To the best of our knowledge, this study is the first to identify latent patterns of shift work among nurses using a large, representative sample from Anhui Province and to examine their associations with comorbid anxiety and depression. Our findings revealed significant differences in comorbid anxiety and depression across the identified shift subgroups. Specifically, nurses in the low‐frequency, high‐seniority group (Class 3) and the high‐frequency, high‐seniority group (Class 1) had statistically significantly higher risks of comorbid anxiety and depression than those in the low‐frequency, low‐seniority group (Class 2). Furthermore, the subgroup analysis results indicated that there was no significant modifying effect on sleep quality.

The results of this study showed that the prevalence of anxiety symptoms and depressive symptoms among the 2791 nurses was 60.5% and 65.1%, respectively. These findings are consistent with those of the Chinese Nurse Health Cohort conducted by Shandong University [6] but slightly higher than those reported in another Chinese study [34] and a study conducted among nurses in tertiary hospitals in South Korea [35]. This discrepancy may be attributed to differences in the survey instruments used as well as the fact that the sample in this study consisted exclusively of female nurses. Compared to males, female nurses have a higher prevalence of mental health issues [36, 37], which may be related to factors such as hormonal secretion and circadian clock gene regulation [38, 39]. In this study, 53.9% of nurses presented with comorbid anxiety and depressive symptoms, a prevalence substantially higher than that reported in other populations [7, 40], indicating that anxiety–depression comorbidity is highly prevalent among nurses. Anxiety and depressive symptoms frequently co‐occur. Compared with individuals with either anxiety or depression alone, those with anxiety–depression comorbidity typically experience more severe clinical symptoms, higher suicide rates, poorer prognosis, and greater impairment in social functioning, often resulting in a heavier public health burden [41, 42].

Consistent with previous studies, the low‐frequency, high‐seniority group (Class 3) was associated with comorbid anxiety and depression, suggesting that cumulative years of shift work exposure, rather than the current monthly frequency alone, may be a critical determinant of poor mental health. A meta‐analysis including seven studies indicated that shift work is associated with an increased overall risk of adverse mental health outcomes [43]. Although nurses in this group worked fewer night shifts per month, long‐term cumulative shift work may have led to progressive disruption of the sleep–wake cycle, with insufficient recovery of accumulated “sleep debt” during nonwork days. Unlike younger nurses, older or more senior nurses often experience more pronounced age‐related declines in circadian adaptability and sleep efficiency, making recovery more difficult even after occasional night shifts. Furthermore, senior nurses typically bear heavier managerial responsibilities, clinical teaching duties, and emotional labor during shifts, which impose significant chronic psychosocial stress that interacts with circadian disruption to further precipitate anxiety and depression. The most concerning finding was the significantly elevated risk of comorbid anxiety and depression among nurses in the (Class 1). Due to long‐term exposure to a high‐stress work environment, these nurses develop irregular sleep patterns, which subsequently lead to various physical and mental health problems [31]. A possible mechanism underlying this phenomenon is that shift work leads to circadian rhythm disruption and dysregulation of the sleep–wake cycle [5, 19]. Shift work schedules induce circadian misalignment. Following night shifts, workers tend to fall asleep quickly upon returning home during the day but are often prematurely awakened by their internal biological rhythms, resulting in pronounced daytime sleepiness and mental fatigue [5, 21]. Additionally, the high‐stress nature of nursing work frequently places nurses in a state of heightened tension, which further compromises their mental health [4, 44]. The low‐frequency, low‐seniority group (Class 2) exhibited the most favorable mental health outcomes. However, this does not imply complete immunity. Younger nurses in the early phase of shift work may still possess greater circadian flexibility and physiological resilience, and their shorter cumulative exposure limits the long‐term allostatic load. Nevertheless, this group should be monitored prospectively as they are at risk of transitioning into higher‐risk patterns as their tenure increases.

In subgroup analyses, we used sleep quality as a stratification factor. Our findings indicate that although the interaction test did not reach statistical significance, suggesting no significant moderating effect of sleep quality on the outcomes, stratified analyses revealed a significant association between Class 1 and anxiety levels only among nurses with good sleep quality, whereas no such association was observed among those with poor sleep quality. These results suggest that while sleep quality does not statistically interact with shift work patterns, there may be differences in the detectability or strength of the association across subgroups. Future studies with larger sample sizes are warranted to further explore potential subgroup differences.

To our knowledge, this is the first study to explore the association between three latent classes of shift work and comorbid anxiety and depressive symptoms in a large‐scale female nurse population. These findings have important implications for clinical practice among nursing staff and for prevention strategies for comorbid anxiety and depression. First, interventions should be tailored to specific shift groups. Priority in specialized psychological support services or flexible mental health leave systems should be given to nurses engaged in long‐term, high‐stress shift work. Managers should implement mandatory “circadian recovery breaks,” such as a minimum of 48 consecutive hours off after three to four consecutive night shifts, to facilitate the resetting of the sleep–wake cycle. For nurses in the low‐frequency, high‐seniority shift work group, hospital administrators should consider age‐based scheduling priorities, for example, exempting older nurses from night shift duties or limiting their role to “on‐call” rather than full nocturnal shifts. For the low‐frequency, low‐seniority group, managers should avoid imposing excessive workloads prematurely. Instead, a structured adaptation plan should be implemented, such as gradually increasing the night shift frequency over the first 2 years rather than assigning full rotation immediately. Prior to these nurses transitioning into high‐risk work patterns, pairing them with senior mentors, providing psychological support, and offering training in sleep hygiene and emotion regulation skills can serve as primary preventive strategies. Second, organizational strategies, such as encouraging nurses to proactively report psychological distress, fostering a supportive professional atmosphere, and reducing the stigma associated with seeking mental health help, may enhance the effectiveness of individual‐level interventions [45]. The main limitation of this study lies in its cross‐sectional design, which limits the conclusiveness of causal inferences regarding the relationships among shift work and anxiety–depressive symptoms. In addition, the possibility of selection bias cannot be completely ruled out in this study. On one hand, nurses who were overworked and emotionally exhausted might have been less likely to respond due to time or energy constraints, leading to nonresponse bias; on the other hand, individuals with higher levels of psychological distress might have been more motivated to participate in a survey on mental health, thereby introducing self‐selection bias. Furthermore, the use of electronic questionnaires to collect information on shift work, mental health, sleep quality, and general variables may have introduced a recall bias. Finally, although this study comprehensively considered relevant covariates such as marital status, number of children, demographic information, and health‐risk behaviors among female nurses, unadjusted covariates may still influence the robustness of the findings.

## 5. Conclusion

This study highlights the importance of recognizing shift patterns in understanding nurses’ mental health status. The high‐frequency, high‐seniority group exhibited the highest risk of anxiety and depression, indicating the need for multifaceted, targeted interventions. Developing individualized support programs tailored to specific shift patterns holds promise for improving nurses’ mental health and thereby contributing to broader health promotion goals.

## Author Contributions

**Chen Zhao:** writing – review and editing, writing – original draft, visualization, methodology, formal analysis, conceptualization, investigation. **Xuezhi Shi, Min Huang, Meng Fan, Miao Zhang, Wendi Xu, Yapeng Deng, Mei Jin, Xinyu Hu, and Heping Wang:** investigation. **Yajuan Yang:** data collection, funding acquisition. **Yaqian Niu:** writing – review and editing, writing – original draft, investigation. **Ying Tang and Minmin Jiang:** supervision, funding acquisition. **Fangbiao Tao:** supervision. **Xiaoyan Wu:** writing – review and editing, supervision, methodology, funding acquisition, conceptualization.

## Funding

This work was supported by the National Natural Science Foundation of China (Grant 82173542), the Graduate Youth Cultivation Project of School of Nursing, Anhui Medical University (Grant hlpy20210009), the National Natural Science Foundation of China (Grant 82504429), the Natural Science Foundation of Anhui Province (Grant 2408085QH278), and the Nursing Project of Anhui Institute of Translational Medicine (Grant 2025zhyx‐hl‐A08).

## Conflicts of Interest

The authors declare no conflicts of interest.

## Data Availability

The data that support the findings of this study are available upon request from the corresponding author. The data are not publicly available due to privacy or ethical restrictions.
